# Central Pressure Appraisal: Clinical Validation of a Subject-Specific Mathematical Model

**DOI:** 10.1371/journal.pone.0151523

**Published:** 2016-03-24

**Authors:** Francesco Tosello, Andrea Guala, Dario Leone, Carlo Camporeale, Giulia Bruno, Luca Ridolfi, Franco Veglio, Alberto Milan

**Affiliations:** 1 Department of Medical Sciences, Division of Internal Medicine, Hypertension Unit, University Hospital ‘AOU Città della Salute e della Scienza di Torino', University of Torino, Torino, Italy; 2 DIATI, Politecnico di Torino, Turin, Italy; Kurume University School of Medicine, JAPAN

## Abstract

**Introduction:**

Current evidence suggests that aortic blood pressure has a superior prognostic value with respect to brachial pressure for cardiovascular events, but direct measurement is not feasible in daily clinical practice.

**Aim:**

The aim of the present study is the clinical validation of a multiscale mathematical model for non-invasive appraisal of central blood pressure from subject-specific characteristics.

**Methods:**

A total of 51 young male were selected for the present study. Aortic systolic and diastolic pressure were estimated with a mathematical model and were compared to the most-used non-invasive validated technique (SphygmoCor device, AtCor Medical, Australia). SphygmoCor was calibrated through diastolic and systolic brachial pressure obtained with a sphygmomanometer, while model inputs consist of brachial pressure, height, weight, age, left-ventricular end-systolic and end-diastolic volumes, and data from a pulse wave velocity study.

**Results:**

Model-estimated systolic and diastolic central blood pressures resulted to be significantly related to SphygmoCor-assessed central systolic (r = 0.65 p <0.0001) and diastolic (r = 0.84 p<0.0001) blood pressures. The model showed a significant overestimation of systolic pressure (+7.8 (-2.2;14) mmHg, p = 0.0003) and a significant underestimation of diastolic values (-3.2(-7.5;1.6), p = 0.004), which imply a significant overestimation of central pulse pressure. Interestingly, model prediction errors mirror the mean errors reported in large meta-analysis characterizing the use of the SphygmoCor when non-invasive calibration is performed.

**Conclusion:**

In conclusion, multi-scale mathematical model predictions result to be significantly related to SphygmoCor ones. Model-predicted systolic and diastolic aortic pressure resulted in difference of less than 10 mmHg in the 51% and 84% of the subjects, respectively, when compared with SphygmoCor-obtained pressures.

## Introduction

Increased blood pressure (i.e., Arterial Hypertension) represents a major cardiovascular risk factor for western populations [1]. Blood pressure (BP) is usually measured at brachial artery, but current evidence suggest that central blood pressure is more strictly related to cardiovascular events [2–4]. Central blood pressure differs from the brachial one because pressure waveform evolves and modifies when it travels along the arterial tree depending on the interaction of a large number of forward and backward waves reflected at multiple sites [5]. It follows that pressure waveforms at different locations vary in both shape and extreme values.

Several studies focused on the prognostic value of central pressure and on its link with brachial pressure. Clinical studies highlighted how systolic and pulse central pressure are more tightly related to carotid artery hypertrophy, coronary occlusion, kidney dysfunction, atherosclerosis extent, left-ventricular hypertrophy, diastolic dysfunction, and reduced ejection fraction than their brachial counterparts [2–4].

However, direct measurement of central blood pressure is not feasible in everyday clinical practice, being a complex, risky procedure for patients [5]. Invasive evaluation consists in fact in inserting a fluid-filled catheter in the femoral artery and moving it through the abdominal and thoracic aorta until reaching the ascending aorta. Aiming to overcome this risk, non-invasive approaches have been developed. New instruments are able to estimate central blood pressure from measurements of peripheral values through applanation tonometry, which are elaborated by transfer functions. Nevertheless, the accuracy of such evaluations has been questioned [5,6]. Indeed, the well-known overestimation of diastolic and underestimation of systolic intra-arterial peripheral pressure obtained through oscillometric device as well as the unconsidered effect of pulse pressure amplification can entail an overestimation of the central pulse pressure evaluation [5,6,7].

Recent, remarkable developments in cardiac imaging techniques and in computing capacity give the opportunity to explore the cardiovascular system through the use of fluid-mechanical mathematical models, which have proven to be capable of reliably describe the main characteristics of the cardiovascular system [8, 9, 10, 11]. In particular, multi-scale models—most of them relying on a one-dimensional description of the wave propagation through the large arteries and on a simpler zero-dimensional characterization of the left ventricle, aortic valve and microcirculation—are now being used in a number studies. E.g., to analyze the pressure waveforms [9, 12], the coronary function [9, 13], and the effect of the aortic shape [11].

In this picture, the aim of the present study is the clinical validation of a mathematical model for the appraisal of the central blood pressure starting from non-invasively obtained subject-specific characteristics [9]. The model is compared with the most used, non-invasive validated tool for the central pressure estimation in the clinical practice (SphygmoCor, AtCor Medical, Sydney, Australia). Model inputs comprises brachial pressure, height, weight, age, left-ventricular end-systolic and end-diastolic volumes, and carotid-femoral, carotid-radial and femoral-tibial pulse wave velocity (PWV).

## Methods

### Study population

A total of 51 young male (S1 File), free of any history of cardiovascular disease and found healthy at a routine clinical and echocardiographic evaluation, were prospectively selected for the present study. Every individual underwent non-invasive evaluation of central and peripheral blood pressures, a pulse wave velocity study and a complete transthoracic echocardiogram. Subjects with blood pressure values higher than 140/90 mmHg were excluded; whenever pressure values were ambiguous, 24-hour blood pressure monitoring measurements were performed. The study has been evaluated and approved by our local ethic committee (Comitato Etico Interaziendale A.O.U. Città della Salute e della Scienza di Torino—A.O. Ordine Mauriziano—A.S.L. TO1—CEI/330). All subjects provided their written informed consent to participate in this study.

### Clinical evaluation

All enrolled individuals underwent complete clinical evaluation with extensive anamnestic record and clinical examination. Height and weight were recorded. Peripheral brachial blood pressure was measured with a standardized approach following current international guidelines [1] with oscillometric validated device (Omron Matsusaka, Kioto, Japan). Tonometric measurements were started after stabilization of brachial pressure values was obtained with an appropriate resting period. Brachial arterial pressure was checked before every tonometric evaluation.

### Evaluation of central blood pressure by the SphygmoCor device

The central hemodynamic measurements employed in this study have been previously validated [14,15]. Radial artery waveforms were obtained with a high-fidelity micromanometer (SPC-301; Millar Instruments, Houston, TX, USA) from the wrist and a corresponding central waveform was generated with a generalized transfer function (SphygmoCor, AtCor Medical, Sydney, Australia), which has been widely validated by using invasive measurements of radial waveforms [14,15]. Calibration of the radial arterial waveform obtained by applanation tonometry was carried out with systolic and diastolic blood pressure values recorded non-invasively on the contralateral side using a validated automatic oscillometric device. This evaluation of central blood pressures was carried out simultaneously with echocardiographic acquisition of left ventricular dimensions and outflow flow velocity. Thus, central pressure data and echocardiographic parameters were recorded with no variation on heart rate and hemodynamic conditions.

### Carotid-femoral pulse wave velocity (cfPWV) measurement

Aortic stiffness was obtained following current recommendations [16]. cfPWV, a classic index of arterial stiffness, was measured along the descending thoracic-abdominal aorta by the foot-to-foot velocity method, as previously published and validated [16]. Briefly, waveforms were obtained transcutaneously over the common carotid artery and the right femoral artery, and the time delay was measured between the feet of the two waveforms. The distance covered by the waves was assimilated to the distance measured between the two recording sites. The PWV was calculated as the ratio between distance and time delay using the SphygmoCor system.

### Transthoracic Echocardiography

A two-dimensional echocardiogram was performed at rest in the left lateral decubitus position with commercially available ultrasound systems equipped with tissue Doppler imaging software (Philips iE33, Philips Healthcare, Eindhoven, The Netherlands). Multiple frequency phased array transducers (2–4 MHz) were used. Technical details have been previously reported [17]. In the present work, we focused on left ventricular end systolic and end diastolic volumes, which are measured using Simpson biplane method and when possible, 3D echocardiography, following current international recommendations [18].

### Evaluation of central pressure by the mathematical model

The mathematical model used for central pressure appraisal has been previously presented and described [9,12,13]. Blood flow/pressure dynamics in the large artery network are described by one-dimensional modeling, which includes the non-linear viscoelasticity of the arterial walls, while left ventricle, its valves and distal microcirculation are described by lumped models. The mathematical model is tailored on each individual by a subject-specific setting procedure, which has been designed and verified using data from 6 healthy volunteers, with clinical and hemodynamic characteristics similar to those of the subjects here included. Starting from non-invasive subject’s characteristics, a number of model parameters are modified through a subject-specific setting procedure. They concern large-arteries geometrical and mechanical characteristics, like vessel diameters, lengths and pulse wave velocity, and descriptors of the left ventricular activity, e.g. ventricular elastance, heart rate and activation time. The only improvements with respect to the subject-specific setting procedure described in our previous works concern (i) the use of body surface instead of body mass index for the body size characterization and (ii) the refined quantification of the distal model resistance. This latter is obtained first by quantifying the subject-specific mean pressure-flow ratio, where the mean pressure is assessed from systolic and diastolic brachial pressure values using the 2/3 approximation, while the mean flow is obtained multiplying stroke volume per heart pacing. The subject-specific ratio is then referred to the mean pressure-flow ratio calculated using the reference parameters as a model input, obtaining a coefficient which is used to multiply the reference distal resistance values and thus to set the subject-specific distal resistances.

These improvements entail that the subject-specific setting procedure is totally automatic.

### Statistical analysis

Statistical analysis was conducted using SAS V9.1 software (SAS Institute Inc.–Cary, NC, USA) and using custom-designed software written in Matlab (The Mathworks, Natick, MA). The parametric distribution of the variables was analysed using the Kolmogorov Smirnov test and residual analysis. Data are expressed as mean ± Standard Deviation (SD) or as median and interquartile difference if appropriate. Differences between means were examined using a t test or ANOVA for normal distributed variables. Kruskal Wallis or non-parametric ANOVA were used for non-normally distributed variables. Linear regression analysis was generated between mathematical model- and SphygmoCor-derived central pressure values. The calculated central blood pressure values were then analysed using Bland-Altman analysis, in order to assess the agreement between the two methods. Statistical significance was assumed if the null hypothesis could be rejected at p<0.05.

The existence of possible statistically significant links between errors and input data was investigated with a multivariate regression among pressures and subjects characteristics used as input, while a communality analysis [19] is further applied to rank these dependences. A correlation analysis is performed to uncover subject characteristics that could predict differences between model and SphygmoCor estimates for both systolic and diastolic values.

## Results

Clinical and hemodynamic features of the study population are summarized in Table 1.

**Table 1:** Clinical characteristics of the study population. BMI = body mass index; HR = heart rate; bSBP = brachial systolic blood pressure; bDBP = brachial diastolic blood pressure; ESV = LV end-systolic volume; EDV = LV end-diastolic volume; SV = stroke volume.

Age (years)	24.3±1.59
Height (cm)	178.±6.15
Weight (Kg)	75±10.2
BMI (Kg/m^2^)	23.5±2.5
HR (bpm)	62.1±11.15
bSBP (mmHg)	121±12.1
bDBP (mmHg)	67.4±8.73
ESV (cm^3^)	53.57±8.97
EDV (cm^3^)	131.1±21.3
SV (cm^3^)	82.02±19.73
cfPWV (m/s)	5.86±0.87

In Table 2 mean values for systolic and diastolic central blood pressure computed with the mathematical model and obtained non-invasively with the SphygmoCor were compared.

**Table 2:** Mean and percent differences between the mathematical model and SphygmoCor. cDSP, cDBP and cPP refer to central systolic, diastolic and pulse pressure, respectively.

	Model	SphygmoCor	P	Model—SphygmoCor	Percent difference
cSBP (mmHg)	109±18.6	101.2±9.9	0.0003	7.8±14.3	7.53±14.3
cDBP (mmHg)	64.9±12.3	68.1±9.1	0.004	-3.2±7.7	-4.87±11.3
cPP (mmHg)	44.1±10.5	33.2±5.4	<0.0001	10.9±12.5	20.3±23.1

Differences between model- and SphygmoCor-derived values were overall satisfactory, with a mean difference below two times the standard deviation of the pressure set evaluated with SphygmoCor. The model showed a significant over-estimation of systolic pressure (+7.8 (-2.2; 14) mmHg, p = 0.0003) and a significant under-estimation of diastolic values (-3.2 (-7.5; 1.6) mmHg, p = 0.004), leading to a significant over-estimation of pulse pressure (p<0.0001).

Mean and percent differences of the mean values of estimated central pressures, obtained with the two methods, are summarized in Table 2.

**Fig 1:**
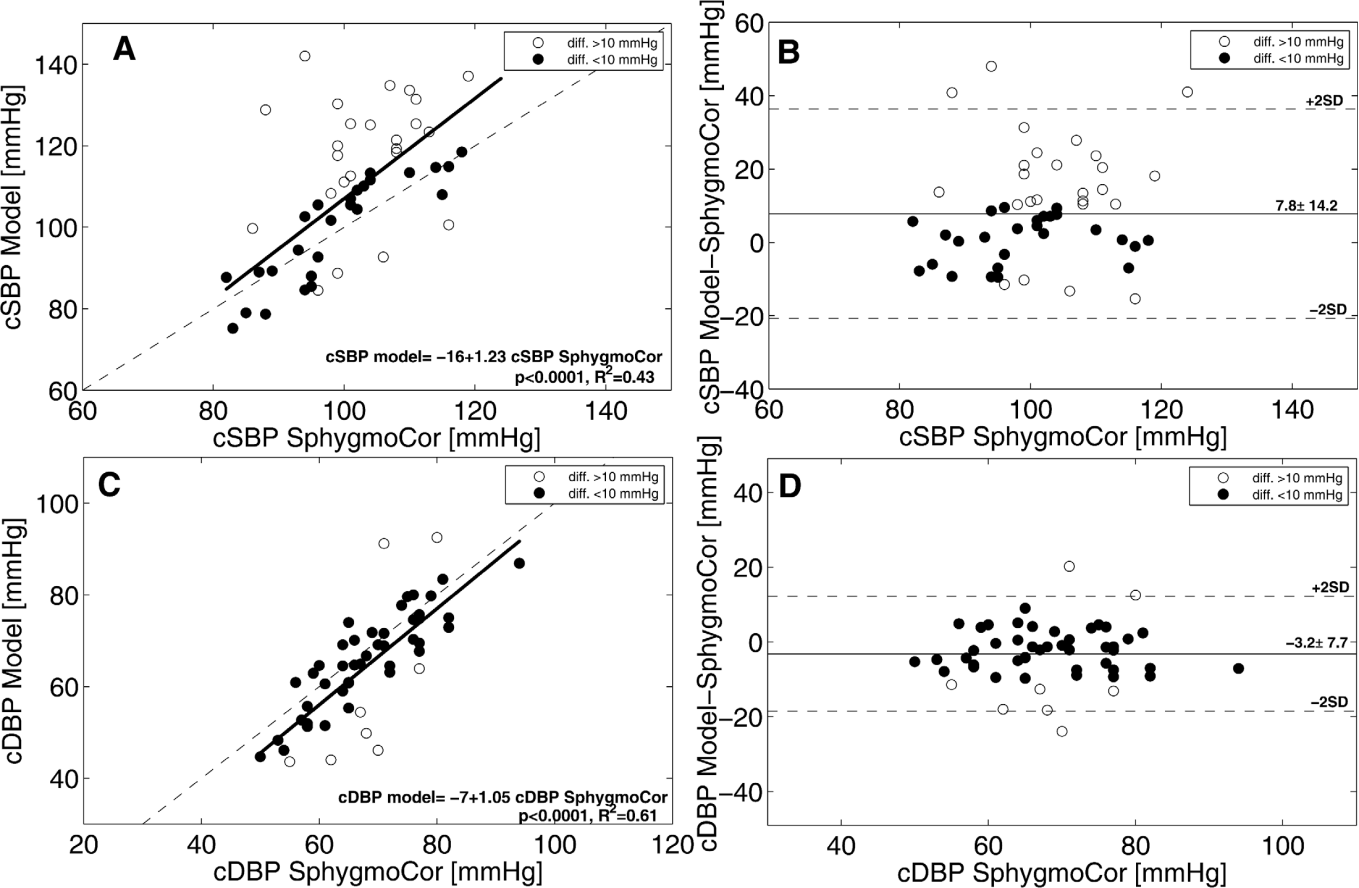
Comparison between systolic (top) and diastolic (bottom) model- and SphygmoCor- derived central pressure values. Linear regression analysis (left) and Bland Altman (right) are reported for both quantities.

A visual representation of the relations between model- and SphygmoCor-derived quantities is provided in Fig 1, where left panels (A and C) depict linear regression analysis while right panels (B and D) report Bland Altman evaluation for systolic (top) and diastolic (bottom) central blood pressure values. Model-derived systolic and diastolic central blood pressure values resulted to be significantly related to Sphygmocor-assessed systolic (r 0.65, p<0.0001) and diastolic (r 0.84, p<0.0001) values. Bland Altman plots further reveals how both error trends tends to increase with increasing pressure.

A threshold of 10 mmHg was set for defining the model-derived appraisal “satisfactory” (i.e. difference in estimated using the model and using SphygmoCor values within 10 mmHg). Overall, 52% of the evaluated subjects had satisfactory model-derived estimations of central systolic pressure, a percentage that rose to 84% for diastolic values.

In order to investigate the existence of possible statistically significant links between errors and input data, a multivariate regression among systolic (and diastolic) pressure and subject characteristics is performed, where t-test (α = 0.025) is used to select significant regressors. Systolic pressure errors result significantly correlated to brachial diastolic (t = -17.1) and systolic (t = 10.8) pressure. Differently, diastolic pressure are linked to carotid-femoral pulse wave velocity (t = 11.1) and diastolic (t = -6.0) and systolic (t = 2.2) brachial pressures. Aiming to ranking these dependences, the communality analysis is used [20]. It reveals that diastolic brachial pressure is the most important parameter (its unique contribute is U_1_ = 0.67) for systolic pressure errors, while cfPWV largely dominates diastolic errors (U_1_ = 0.45).

Finally, a correlation analysis was performed to uncover subject characteristics that could predict differences between model and SphygmoCor estimates for both systolic and diastolic values. Estimation differences between clinical and hemodynamic characteristics where divided in those pertaining to subjects with acceptable and impaired accuracy and are reported in Table 3.

**Table 3:** Clinical and hemodynamic characteristics in subjects with acceptable vs. impaired accuracy in central blood pressure estimation. BSA = body surface Area; CO = cardiac output; CI = cardiac index.

	Central systolic pressure			Central diastolic pressure		
	Acceptable Accuracy	Impaired Accuracy	p	Acceptable Accuracy	Impaired Accuracy	p
**Clinical variables**	n.26	n.25		n. 43	N.8	
**n**	51	49		84.3	15.7	
Height (m)	176.1±5.6	180.0±6.1	0.02	177.4±6.2	181.5±5.0	0.07
Weight (Kg)	72.8±10.1	77.4±1	0.11	74.3±10.4	79.3±8.7	0.2
BMI (Kg/m^2^)	23.3±2.5	23.8±2.5	0.42	23.5±2.4	24.1±2.8	0.51
BSA (m^2^)	1.9±0.1	2.0±0.1	0.06	1.9±0.1	2.0±0.1	0.13
bSBP (mmHg)	119.6±12.4	124.2±11.7	0.18	121.5±12.2	123.4±12.4	0.69
bDBP (mmHg)	63.8±7.9	71.4±7.9	0.001	67.4±9.0	67.6±7.4	0.95
HR (bpm)	61.7±9.7	62.6±12.7	0.76	60.4±10.3	71.7±11.7	0.007
**Hemodynamic variables**						
ESV (cc)	50.95±7.9	56.4±9.3	0.02	53.5±9.3	54.0±7.3	0.89
EDV (cc)	127.2±21.1	135.2±21.1	0.17	131.3±22.6	129.5±13.0	0.82
SV (cc)	81.9±20.1	82.1±19.8	0.95	82.8±20.7	77.5±13.3	0.04
CO (l/minute)	5.2±1.3	4.9±1.1	0.31	5.0±1.2	5.2±1.2	0.63
CI (l/min/m^2^)	2.7±0.6	2.5±0.5	0.10	2.6±6.0	2.6±6.1	0.98
cfPWV (m/s)	5.57±0.78	6.18±0.86	0.01	5.7±0.8	6.5±1.1	0.01

Patients with less accuracy estimations for systolic values have higher brachial diastolic pressure (p = 0.001), are significantly higher (p = 0.02), have larger end systolic left ventricular volume (p = 0.02), and have higher cfPWV (p = 0.01). Diastolic low accuracy estimations are related to higher heart rate (p = 0.007) and higher cfPWV (p = 0.01).

## Discussion

The cardiovascular system is a complex apparatus in which heart, large vessel and their branches closely interact. Forward pressure waves generated by systolic contraction propagate through the arterial tree, and they are partially reflected at every impedance mismatch generating backward propagating pressure waves. These backward propagating waves are further reflected at upstream bifurcation, creating a complex pattern rapidly smoothed out by blood and vessel wall viscosity [12]. Therefore, arterial pressure in a given measurement location results from the sum of the location-specific forward and backward components. It follows that blood pressure measured at the arm (brachial pressure) is consistently different from the one affecting the aorta (central pressures) [5]. Driven by several correlations encountered in a number of clinical studies [2–4], the estimation of central blood pressures gained gradual consent in the scientific community and literature over the past years [18]. Literature data are consistent [14, 21] in indicating central pressure as a better marker of cardiac afterload.

These facts triggered great efforts for the development of non-invasive techniques for the central pressure evaluation. As a result, nowadays a large number of instruments are commercialized for the estimation of central blood pressure, although high costs and low availability still represent major limitations for their widespread utilization. Among various non-invasive techniques, SphygmoCor device is the most widely used in clinical studies [14]. It is based on the application of a generalized transfer function to the tonometric radial pressure waveform, which in turn has to be calibrated. Although this device showed impressive reliability when invasive radial pressure is used as input (−1.1±4.1 mmHg for systolic and −0.5±2.1 mmHg for diastolic values [6]), not negligible errors have been detected when non-invasive calibration is performed [6]. They are due to both the oscillometric evaluation of brachial pressure and the assumption of no pulse pressure amplification between brachial and radial locations. This leads to an overestimation and underestimation of systolic and diastolic central pressures, respectively, when SphygmoCor device is calibrated to cuff blood pressure: a large meta-analysis reported −8.2±10.3 mmHg and 7.6±8.7 mmHg for the systolic and diastolic pressures, respectively [6].

The multi-scale mathematical approach for the cardiovascular system modeling can contribute to overcome these limitations. Recently, mathematical model of the cardiovascular system have indeed proven to be able to provide subject-specific description [8,9], reaching the forefront of central pressure estimation research.

For the first time in literature, the present study proposes the application of a refined mathematical model for the estimation of the central blood pressure in healthy young male, starting from subject-specific non-invasively obtained features. Results allow us to state that the multi-scale model, along with the suitable subject-specific setting procedure, is able to predict with good accuracy (here referred to differences under 10 mmHg with respect to SphygmoCor estimations) systolic central pressure in little more than half of the evaluated subjects (51%), and the diastolic central pressure in almost the entire population (84%). Furthermore, model-derived central systolic pressures result to be generally lower than the SphygmoCor estimations, while diastolic values are higher. Notice that if the SphygmoCor estimations were adjusted to include the bias arising from its non-invasive calibration [6, 7], the errors of the mathematical model would become very small: the mean difference would be equal to 0.4 mmHg for the systolic blood pressure and 4.4 mmHg for the diastolic ones. However, such a bias correction has to be considered as a speculation, as we have no data confirming that errors affecting our central pressure estimations obtained with the SphygmoCor exhibit the same mean value (i.e., the bias) measured in the large scale tests [6, 7]. Moreover, no information about error distribution can be draft, as the covariance between model- and Shygmocor-affecting errors is unknown. However, the favorable comparison of the Bland Altman plots—which shows how errors tend to increase with higher pressures (see Fig 1) as found in clinical studies comparing invasive aortic pressure with SphygmoCor estimations with identical calibration [21], strongly suggests the existence of a non negligible covariance.

In spite of this remarkable improvement of the model accuracy evaluation, we do not feel of supporting this inference, as it is methodologically inadequate. More properly, we prefer concluding that a more accurate validation of the mathematical model will require invasive measurements. This will grant a deeper evaluation of the model soundness as well as giving clues on its applicability in a wider populations.

Central hemodynamic is one of the most innovative and appealing field in the study of physiopathology and treatment of cardiovascular disease. As underlined by current guidelines on Essential Hypertension, research on central hemodynamic is hampered primarily by scant availability of dedicated instruments, still complex and expensive. The development of a mathematical model able to predict accurate central blood pressure values for a single patients, given easily collectable data (age, sex, height, weight, heart rate and ventricular volumes), may thus represent an innovative approach granting wider application of the analysis. In the general population, this may lead to better risk stratification regarding cardiovascular events.

Advantages arising from the mathematical modelling approach concern the concomitant evaluation of a number of hemodynamics quantities. E.g., LV work, coronary flow condition, wave energy, and wave reflections can be retrieved from the model and their potentialities as biomarker can be explored. Moreover, in silico models do not need dedicated, expensive instrumentations, which are the most strong limiting factor to the widespread diffusion of central pressure evaluation, despite the well-known prognostic value of this quantity. Finally, in silico approach can adapt to different scenarios, as in the case of appraisal of post-surgery conditions or when the impact of geometrical and/or mechanical changes (e.g., aneurism-induced) are investigated.

## Limitations

A few limitations of the present study need to be underlined, in order to critically appreciate its results. The present data are so fare relevant only for healthy young male individuals, due to the *a priori* selection criteria. This restrictive choice has been made after careful consideration of available literature, which confirms a good accuracy for estimation of physical properties of great vessel in this specific subset of individuals [8]. In order to generalize these results, the mathematical model will have to be tested on female subjects, on individuals in different age ranges, and on patients with pathologic condition (i.e., hypertension).

Furthermore, the model was tested against a non-invasive estimation of central blood pressures, which represents *per se* an approximation compared to the gold standard of invasive direct recording. Anyway, we adopted the most used instrument in non-invasive clinical studies [14].

## Conclusions

This study demonstrates that the proposed multi-scale mathematical model allows central aortic pressure to be predicted with good accuracy, using few non-invasive measurements, in at least more than half of a population of young healthy male subjects. Moreover, mean central systolic pressure differences largely mirror the systematic error reported when the generalized transfer function is used without invasive calibration. Results encourage to test the proposed mathematical model against invasive measurements of central pressure and on different sets of individuals (i.e., females, patients with pathologies, etc.).

## Supporting Information

S1 File Minimum dataset. (XLS)
