# Correction: Central Pressure Appraisal: Clinical Validation of a Subject-Specific Mathematical Model

**DOI:** 10.1371/journal.pone.0157117

**Published:** 2016-06-03

**Authors:** Francesco Tosello, Andrea Guala, Dario Leone, Carlo Camporeale, Giulia Bruno, Luca Ridolfi, Franco Veglio, Alberto Milan

There are errors in the Author Contributions. The correct contributions are: Conceived and designed the experiments: AM FT AG CC LR. Performed the experiments: FT DL GB AM. Analyzed the data: AM FT AG CC LR. Contributed reagents/materials/analysis tools: FV. Wrote the paper: AM FT AG LR
